# Pathways to Reduced Overnight Hospitalizations: Evaluating 62 Physical, Behavioral, and Psychosocial Factors

**DOI:** 10.1093/geroni/igab046.3423

**Published:** 2021-12-17

**Authors:** Jean Oh, Julia Nakamura, Eric Kim

**Affiliations:** 1 University of British Columbia, Vancouver, British Columbia, Canada; 2 University of British Columbia, University of British Columbia/Vancouver, British Columbia, Canada

## Abstract

As healthcare costs rise steadily and rapidly, researchers and policymakers are increasingly interested in reducing healthcare utilization costs. Growing evidence documents many factors that may influence healthcare utilization; however, less is known about how changes in candidate predictors influence subsequent healthcare utilization. Using data from 11,374 participants in the Health and Retirement Study (HRS)—a diverse, longitudinal, and nationally representative sample of older adults in the United States, we evaluated a large range of candidate predictors of overnight hospitalizations. Using generalized linear regression models with a lagged exposure-wide approach, we evaluated if changes in 62 predictors over four-years (between t0;2006/2008 and t1;2010/2012) were associated with subsequent hospitalizations during the two years prior to t2 (2012-2014 (Cohort A) or 2014-2016 (Cohort B)). After adjustment for a rich set of baseline covariates, changes in some health behaviors (e.g., frequent physical activity), physical health conditions (e.g., no physical functioning limitations), and psychosocial factors (e.g., higher purpose in life, lower anxiety, more volunteering) were associated with decreased hospitalizations four years later. However, there was little evidence that other factors (e.g. smoking, obesity) were associated with subsequent hospitalizations. Notably, some psychosocial factors had effect sizes as large as some physical health conditions. Several indicators of physical health, health behaviors, and psychosocial well-being may predict subsequent hospitalizations, and these factors may be novel targets for interventions and policies aiming to reduce healthcare costs in older adults.

